# Low-Latency and Energy-Efficient Data Preservation Mechanism in Low-Duty-Cycle Sensor Networks

**DOI:** 10.3390/s17051051

**Published:** 2017-05-06

**Authors:** Chan Jiang, Tao-Shen Li, Jun-Bin Liang, Heng Wu

**Affiliations:** 1 School of Electronic and Information Engineering, South China University of Technology, Guangzhou 510641, China; tshli@gxu.edu.cn; 2 School of Computer, Electronics and Information, Guangxi University, Nanning 530004, China; liangjb@gxu.edu.cn; 3 Department of Computer Science, College of Engineering, Texas Tech University, Lubbock, TX 79409, USA; heng05.wu@ttu.edu

**Keywords:** low-duty-cycle sensor networks, data preservation, data dissemination, latency, energy efficiency

## Abstract

Similar to traditional wireless sensor networks (WSN), the nodes only have limited memory and energy in low-duty-cycle sensor networks (LDC-WSN). However, different from WSN, the nodes in LDC-WSN often sleep most of their time to preserve their energies. The sleeping feature causes serious data transmission delay. However, each source node that has sensed data needs to quickly disseminate its data to other nodes in the network for redundant storage. Otherwise, data would be lost due to its source node possibly being destroyed by outer forces in a harsh environment. The quick dissemination requirement produces a contradiction with the sleeping delay in the network. How to quickly disseminate all the source data to all the nodes with limited memory in the network for effective preservation is a challenging issue. In this paper, a low-latency and energy-efficient data preservation mechanism in LDC-WSN is proposed. The mechanism is totally distributed. The data can be disseminated to the network with low latency by using a revised probabilistic broadcasting mechanism, and then stored by the nodes with LT (Luby Transform) codes, which are a famous rateless erasure code. After the process of data dissemination and storage completes, some nodes may die due to being destroyed by outer forces. If a mobile sink enters the network at any time and from any place to collect the data, it can recover all of the source data by visiting a small portion of survived nodes in the network. Theoretical analyses and simulation results show that our mechanism outperforms existing mechanisms in the performances of data dissemination delay and energy efficiency.

## 1. Introduction

Wireless sensor networks (WSN) are kinds of ad hoc networks, which are always deployed in harsh environments and with large amounts of autonomous nodes to perform specified tasks, such as environment monitoring and target tracking. Each node in WSN only has limited energy and storage space (memory), so it is important to carefully design data transmission and preservation strategies under these limitations. Low-duty-cycle sensor networks (LDC-WSN) are a new form of WSN [1], where duty-cycle represents the proportion of node’s awaking time *t* in a working period *T*, i.e., *t*/*T*. Low-duty-cycle means that each node’s duty-cycle is lower than 1/3. In an LDC-WSN, the nodes keep sleeping most of the time. Therefore, they can effectively save their energy and work for a very long period, i.e., network lifetime of LDC-WSN is much longer than that of traditional WSN, which benefits the reductions of network deployment cost and maintenance cost [2]. 

However, the sleeping feature of nodes causes serious data transmission delay. In each transmission, a sender node often has to wait for some time until a receiver node wakes up. Therefore, the total latency for a source node to disseminate its data to the whole network is extremely large. Furthermore, the latency would become worse when the network communication is unreliable. 

On the other hand, each source node that has sensed data always wants to disseminate its data to other nodes in the network for preservation as quickly as possible. This is because the source node may be suddenly destroyed by outer forces (e.g., earthquake, flood) and lose its data. The quick dissemination requirement produces a contradiction with the large dissemination delay in the network. 

It is a challenging issue that all of the source data can be quickly disseminated to all nodes in LDC-WSNs. Moreover, it is a challenge that the data can be effectively stored at the nodes with limited memory. Our objective is that all of the data can be recovered even if some nodes die after the network works for a period of time. 

In this paper, a low-latency and energy-efficient data preservation mechanism named FDP (Fast Data Preservation) is proposed. It considers the sleeping feature of the nodes in LDC-WSN and uses a revised probabilistic broadcasting mechanism to disseminate the data. When the data is received by the nodes in the network, they will be stored by using the LT codes. FDP is a totally distributed mechanism, and it needs no any global information such as network topology and geographical locations of the nodes. Each node will make its decision about whether or not to relay its received data according to the number of its neighbors and its energy level. After the process of data dissemination and storage is completed, a mobile sink can enter the network from any place and at any time. The sink will recover all of the source data after it visits a proportion of survived nodes and collects the encoded data stored at the memories of these nodes. To the best of our knowledge, this paper is the first one that researches how to achieve effective data preservation in LDC-WSN. 

The rest of the paper is organized as follows. In Section 2, we will describe related work. The system model and problem statement will be introduced at Section 3. In Section 4, basic idea and detailed description of our algorithm are presented. In Section 5, simulations and evaluations are performed. Finally, we conclude the paper in Section 6.

## 2. Related Work 

Recently, there have been many works focused on data preservation in WSN. They mainly researched how to preserve the data in the nodes with limited memories and achieve some kind of network resilience. According to their ways of preserving the data, existing works can be divided into two classes: feature projection based mechanisms and network coding based mechanisms.

### 2.1. Feature Projection Based Mechanisms

CDP (Compressive Data Persistence) [3] firstly uses random walks [4] to disseminate the data, where the random walks mean that each node would randomly choose one of its neighbors as a receiver in each transmission. When a node receives data, it will compute a value of random linear projection [5] on the data and then save the value. The value needs less memory, compared with the data. CDP has a high decoding ratio, i.e., all of the source data can be recovered by only acquiring a small number of the projection values. However, the random walks produce too many transmissions, which cause large latency in the network and heavy energy consumption at the nodes.

CNCDS (Compressed Network Coding based Distributed data Storage) [6] considers energy efficient performance of the data dissemination and then proposes an adaptive data dissemination based data preservation mechanism. In the process of data dissemination, each node will choose a proper probability to relay data according to the number of its neighbors. All of the data can be received by all of the nodes in the network with low latency and low energy consumption.

CDP and CNCDS only consider how to use a node’s spatial correlation to compute the projection, and do not consider the temporal correlation among the nodes. ST-CNC (Spatio- Temporal Compressive Network Coding) [7] considers the spatial-temporal correlation among the nodes and then redesigns the rules of data projection. It achieves a higher decoding ratio. 

P-STCNC (Practical Spatio-Temporal Compressive Network Coding) [8] considers not only the spatial-temporal correlation, but also the situation that the data is sparse in real networks. Then, a new data dissemination mechanism that can adaptively adjust the communication footstep is proposed. P-STCNC achieves lower energy consumption than ST-CNC.

In general, the feature projection based mechanisms can achieve a high decoding ratio, which benefits the recovery of the data. However, they need complex matrix computations, which are difficult to perform at the nodes with limited computation ability. Moreover, they require that the data has high correlation, which is not suitable for the applications for which the data has little correlation. Therefore, it is difficult for these mechanisms to be widely applied.

### 2.2. Network Coding Based Mechanisms

Network coding based mechanisms have comparatively lower computation complexity, and they are more suitable for being used in the network composed of nodes with limited computation ability. Moreover, they do not require data correlation. Therefore, they have wider application range. According to the types of codes, existing works can be classified into two categories: random linear codes based mechanisms and fountain codes based mechanisms. In random linear codes [9], each encoded piece of data is the random linear combination of several sources of data in a finite field. In fountain codes [10], a set of source data can produce infinite encoded data, and all of the source data can be recovered as long as the users get a set of encoded data whose number is equal to or a little larger than the number of the source data. Next, we will introduce the existing works of the two categories in detail. 

#### 2.2.1. Random Linear Codes Based Mechanisms

PRLC (Priority Random Linear Codes) [11] decides its coding degree according to the importance of the data, where the coding degree is the number of source data used to forms the linear combination. It assigns the data with higher importance with a lower coding degree. By this way, the sink can recover the importance data after it collects lesser encoded data. On the other hand, the data with lower importance will be assigned a higher coding degree, so the information volume in each node’s memory and the use ratio of the memory are increased. However, the data with lower importance need more encoded data to be recovered.

RLC (Random Linear Coding) [12] is focused on the application that the network is deployed in a dangerous field that even a mobile sink cannot visit. In the network, the nodes have to exchange their encoded data with their neighbors continuously. Finally, the encoded data is disseminated to the border of the network. The sink can collect the encoded data at the border and need not enter the network. However, the nodes at the border are required to store large amounts of data, which is not practical for the nodes that only have a limited memory.

DEC-EaF (Decentralized Erasure Codes based Encode-and-Forward) [13] considers the energy efficiency of data dissemination. Each node chooses a target node to receive its data, which avoids the use of random walks. In the process of data transmission, relay nodes would encode and store the data. However, DEC-EaF requires that the source nodes know the geographic information of all other nodes in the network, which is also not practical. 

RRA (Robust Randomized Algorithm) [14] assumes that there are two classes of nodes in the network: normal nodes and strong nodes. A normal node’s memory only has one unit of space, and a strong node’s memory has multiple space units. Each normal node can randomly choose several strong nodes and send its data to them. If a strong node receives the data from a normal node, it will encode the data using random linear codes and then store the encoded data. RRA can achieve high decoding ratio, but its scalability is low for it requires the existence of the strong nodes.

#### 2.2.2. Fountain Codes Based Mechanisms

EDFC (Exact Decentralized Fountain Codes) [15] firstly requires the source nodes to disseminate multiple copies of their data to the network by using random walks. The copies will be saved temporally by some nodes that receive them after they have been transmitted for given numbers of times. After the data dissemination, each node will encode the data copies received according to LT codes. EDFC is not energy efficient, for it uses random walks to disseminate the data copies. Moreover, it requires each node to have a large memory space to save the copies, which is not suitable for the networks in which the nodes only have limited memories. 

ECPC (Erasure Coding with randomized Power Control) [16] improves EDFC by using probabilistic broadcast to disseminate the data directly and not the copies. The nodes can directly encode the data received and need not temporally save them. Therefore, ECPC has the advantages of low data dissemination latency, high energy efficiency and low memory requirement. However, it requires a communication radius of the nodes to be large enough to cover almost the whole network. Moreover, it needs to know some global information such as the number of source nodes, which is hard to be acquired in practical applications. 

LTDC (LT codes based Distributed Coding) [17] improves ECPC by using multicast to disseminate the data, which lowers the redundancy of data reception and transmission in broadcast and achieves higher energy efficiency. However, it still requires global information such as the number of source nodes in the network. 

DDSLT (Distributed Data Storage based on LT codes) [18] needs no global information. It uses random walks to disseminate data, and the nodes can calculate the number of source nodes by receiving the data. It achieves the same encoding ratio with EDFC, but the energy consumption of data dissemination is much larger than ECPC and LTDC.

APBDP (Adaptive Probabilistic Broadcast-based Data Preservation) [19] considers the factors of energy consumption, latency and global information requirement in data dissemination. Firstly, it uses the Extrema Propagation technology [20] to get the information of total number of source nodes. Then, it adopts probabilistic broadcast to disseminate data to the nodes in the network for encoding and storage. Only a small part of the nodes in the network need to relay the data, which benefits the data dissemination to achieve low latency and low energy consumption. 

However, the above mechanisms are mainly based on traditional wireless sensor networks, in which the nodes would not sleep to save their energies. Therefore, they cannot perform well in LDC-WSN and new mechanisms for LDC-WSN should be designed.

## 3. System Model and Problem Statement

### 3.1. System Model

Assume the network is deployed in a square field *A* of *M* × *M*, in which *n* nodes are randomly distributed. Each node’s communication radius is *r*, i.e., two nodes can communicate with each other, if the distance between them is smaller or equals *r*. All of the nodes form a connected network *G*(*V*, *E*), where *V* = {*v*_1_, *v*_2_,…, *v*_*n*_} is the set of the nodes and *E* is the set of communication edges among the nodes. The nodes perform periodic tasks, such as environment surveillance or hazard monitoring. The working period of the nodes is divided into cycles. 

Similar to [1,2], we assume that each cycle is composed of *m* time units {*t*_1_, *t*_2_,…, *t*_*m*_}. A node only wakes up in one of the time units, and sleeps in all of the other time units if it has no data to transmit. The waking time unit of a node is chosen randomly, and it would not be changed. A node can only receive data in its waking time unit but can wake up to transmit data when its neighbors wake up. A node can achieve local synchronization with its neighbors and know all the waking time units of its neighbors. If a node needs to transmit more than two data packets, it will save the data in a FIFO (First Input First Output) queue for transmission in sequence.

The powers of data transmission and reception are fixed. Each node will consume *E*_s_ energy in transmitting 1-bit data, and it will consume *E*_r_ energy in receiving 1-bit data. This assumption is made only for easy analysis, and our mechanism can be used in the network that the powers of data transmission and reception are adjustable. On the other hand, the links among the nodes are unreliable, and the successful ratio of data transmission on each link is unknown. When a node receives a data packet, it will send back an ACK (Acknowledgement) message to the sender for conformation. Only once the sender node gets all the conformation of its neighbors, does it stop transmitting data. 

Different nodes should have different storage capacities. However, in order to make our mechanism applicable in the worst case, we assume that each node has the minimum storage capacity, i.e., each node can store 1 piece of data. If there are multiple pieces of data that need to be stored, a node must perform an XOR (eXclusive OR) operation to combine them into an encoded data. The size of each data (or encoded data) is *d* bits. Each data *x*_i_ will be encapsulated into a packet *packet*(*x*_i_) for transmission. 

The network needs to periodically collect the data, and its working period is divided into rounds. A round is defined as the maximal time duration that all data can be collected by the sink. We mainly consider the data preservation in a round. At the start of each round, the number of nodes in the network is *n*. In different rounds, there may have different numbers of nodes, for some nodes may die at former rounds. In each round, there are three phases:

(1) *Data production phase*. In this phase, all of the nodes monitor the field, and *k* ≤ *n* nodes sensed the data, i.e., there are *k* source nodes and *k* source data. The source nodes will disseminate a short message that contains its ID to the network to let other nodes know of its existence. After all source nodes disseminate their short messages, all of the nodes in the network know the number *k* of source nodes (or source data).
(2) *Data dissemination and storage phase*. In this phase, the *k* source nodes will disseminate their data to the network for storage. Since each node can only store one piece of data, the node will encode the data received before storing it.
(3) *Data collection phase*. In this phase, the nodes wait for the coming of the mobile sink. Some nodes may die due to being broken by outer force. A mobile sink can enter the network from any place in the network and at any time in this phase. It should visit some survival nodes to collect their encoded data. The collected encoded data is expected to recover all of the source data. On the other hand, if a node senses new data, it can continue to disseminate its data to the network for storage. However, since this dissemination cannot be guaranteed to be completed before the coming of the sink, these data cannot be guaranteed to be recovered. The problem of how to store the new data or update the old data is outside the scope of our research, so we will research it in the future and not discuss it in this paper.

Time durations of the three phases above should be decided by applications. In a normal situation, the time duration of the third phase is much larger than that of the other two phases. In each round, all data should be collected. Therefore, a round should contain enough cycles for the three phases.

### 3.2. Problem Statement

According to the system model, the nodes only have limited resources such as energy levels, communication ranges, computation abilities and storage capacities. Moreover, they would sleep for most of their time in each cycle, and they may die due to being broken by outer force in the *data collection phase*. 

Therefore, there are two challenges that exist in each round of data preservation: (1) how can a source node disseminate its message or data to all the nodes in LDC-WSN with low latency in the *data production phase* and the *data dissemination and storage phase*? and (2) how can every node with limited memory save more data in the *data dissemination and storage phase* so that a mobile sink can recover all the source data after visiting as few as possible survival nodes in the *data collection phase*?

Our research objective is to solve the two challenges above. Therefore, our problem is how to design a distributed, energy-efficient and fast data preservation mechanism for which all source nodes can disseminate their messages or data to all the nodes in LDC-WSN with low latency, and then the data can be stored by all the nodes effectively so that a mobile sink can recover them after visiting as few as possible survival nodes.

## 4. Algorithm Description

### 4.1. Basic Idea

In order to reduce the data dissemination latency and improve energy efficiency, we design a new probabilistic broadcast mechanism for LDC-WSN. In the new mechanism, each node will decide whether to become a relay node with specified probability. The probability is computed according to the number of the node’s neighbors and the node’s energy level. If a node decides to relay a packet, it will wake up at the time units that its neighbors wake up and then send its packet to the neighbors. After it sends a packet to a neighbor, it will wait for an ACK message from the neighbor. If it does not receive the ACK message, it will retransmit its packet to the neighbor at the next cycle. If it receives all of the ACK messages from its neighbors, it stops to relay the packet. If a node receives a data packet (not short message) for the first time, it will use LT codes to encode the data and store it. 

In the above idea, the keys for algorithm implementation are how the nodes decide their probabilities of relaying nodes and how can the nodes use LT codes to encode the data. The implementation process will obviously affect the successful ratio of data recovery. Therefore, we need to design it carefully. Next, we will introduce the new probabilistic broadcast mechanism. Then, we present our data preservation mechanism by using LT codes.

### 4.2. Data Dissemination Mechanism

Currently, there are a lot of works focused on the design of probabilistic broadcast in traditional networks. However, they do not consider the feature that nodes would sleep for a long time in LDC-WSN. Therefore, they are not suitable to be used in LDC-WSN. A new probabilistic broadcast method for LDC-WSN should be designed.

**Lemma** **1** [21]. *In a wireless ad hoc network with n nodes, there is a key probability p_c_. If n is large enough and the nodes in the network can rebroadcast a packet with probability p_i_ ≥ p_c_ when they receive the packet for the first time, all nodes in the network can receive the packet when there are no nodes that rebroadcast the packet in the network (i.e., the process of dissemination completes). However, if p_i_ < p_c_, only a few nodes can receive the packet when the dissemination completes*.

This lemma is the analysis result of the percolation theory [22]. It points out that all the nodes in the network can receive a packet if every node can relay the packet with a probability equals or larger than the key probability *p*_c_. However, *p*_c_’s value should be computed according to the topology of the whole network, which is global information, and it is difficult for individual nodes to know it. Therefore, we need to design a method to help the nodes estimate the key probability and then decide their probabilities to relay a packet.

In our mechanism, the nodes with higher energy levels should have higher probability to relay a packet, so as to balance the energy consumption among the nodes to extend network lifetime. Therefore, we set that each node *v*_i_ will relay a packet with probability *p*_i_ = *p*_c_’ + *a* when it receives the packet for the first time, where *p*_c_’ is the estimated key probability, *a* = (*e*_i_ − *avg*)/*avg* is a adjust factor based on node’s energy, and *avg* is the average energy of *v*_i_ and its neighbors. Next, we will analyze how each node *v*_i_ can compute its probability *p*_i_.

Consider a communication graph *G*_c_(*V*_c_, *E*_c_) that uses the network *G* as its base map. *G*_c_ is empty at first. When a node *v*_i_ broadcasts a packet, *v*_i_ is added into *V*_c_. After *v*_i_ sends its packet to its neighbor *v*_j_ successfully, the edge (*v*_i_, *v*_j_) is added into *E*_c_. When the process of data dissemination is completed, *G*_c_ is a sub-graph of *G*. If all nodes receive the packet, *G*_c_ equals *G*.

**Lemma** **2** [23]. *For any sub-graph G_c_ of a connected graph G, G_c_ can equal G with a probability close to 1 if every node v_i_’s degree d_i_ in G_c_ is larger than the minimal expected degree E_m_(d) in G*.

According to Lemma 2, all nodes in the network can receive a packet, if there are at least *E*_m_(*d*) neighbors that receive the packet at each time of transmission. Among the neighbors, a proportion of nodes will continue to rebroadcast the packet. The number of rebroadcasting nodes is decided by a key probability, which we need to compute as follows. Therefore, our target is to make every node’s degree *d* in *G*_c_ be larger than *E*_m_(*d*). The target can be formulated as:

(1) $$E(d)\geq E_{m}(d).$$

Since *E*_m_(*d*) = log(*n*)/loglog(*n*) [24], Formula (1) can be changed as:

(2) E(d)≥log(n)/loglog(n).

According the feature of *E*(*d*), i.e., *E*(*d*) = $\sum_{d}dP\left(d\right)$, we have:

(3) $$\sum_{d}dP\left(d\right)\geq \log\left(n\right)/\mathrm{loglog}(n),$$

(4) $$\Rightarrow \sum_{d}d^{2}P\left(d\right)\geq d\log\left(n\right)/\mathrm{loglog}\left(n\right).$$

Since the nodes are randomly distributed in the field, their degrees can be modelled as a Poisson distribution *P*(*d*) = $\sum_{i=d}^{\infty }\left(\begin{array}{c} i\\ d \end{array}\right)p^{d}{\left(1-p\right)}^{i-d}P\left(i\right)$. Therefore, Formula (4) can be changed as:

$$\Rightarrow \sum_{d=0}^{\infty }d^{2}\sum_{i=d}^{\infty }\left(\begin{array}{c} i\\ d \end{array}\right)p^{d}{\left(1-p\right)}^{i-d}P\left(i\right)\geq d\log\left(n\right)/\mathrm{loglog}\left(n\right),\;\Rightarrow \sum_{d=0}^{\infty }P\left(i\right)\sum_{i=d}^{\infty }d^{2}\left(\begin{array}{c} i\\ d \end{array}\right)p^{d}{\left(1-p\right)}^{i-d}\geq d\log\left(n\right)/\mathrm{loglog}\left(n\right),\;\Rightarrow \sum_{d=0}^{\infty }P\left(i\right)\left(ip\left(1-p\right)+i^{2}p^{2}\right)\geq d\log\left(n\right)/\mathrm{loglog}\left(n\right),$$

(5) $$\Rightarrow p^{2}E\left(d^{2}\right)+p\left(1-p\right)E\left(d\right)\geq d\log\left(n\right)/\mathrm{loglog}\left(n\right).$$

For any node *v*_i_, *E*(*d*) can be approximated as the number of its neighbors |*N*(*v*_i_)|, *E*(*d*^2^) can be approximated as |*N*(*v*_i_)|^2^ + |*N*(*v*_i_)| according to the feature of Poisson distribution, and *d* equals the number of its neighbors. We have:

$$p^{2}\left({\left|N\left(v_{i}\right)\right|}^{2}+\left|N\left(v_{i}\right)\right|\right)+p\left(1-p\right)\left|N\left(v_{i}\right)\right|\geq \left|N\left(v_{i}\right)\right|\log\left(n\right)/\mathrm{loglog}\left(n\right),$$

$$p^{2}\left(\left|N\left(v_{i}\right)\right|+1\right)+p\left(1-p\right)\geq \log\left(n\right)/\mathrm{loglog}\left(n\right),$$

(6) $$p^{2}\left|N\left(v_{i}\right)\right|+p\geq \log\left(n\right)/\mathrm{loglog}\left(n\right).$$

Since 0 ≤ *p* ≤ 1, Formula (6) can be approximated as:

$$p^{2}\left|N\left(v_{i}\right)\right|\geq \log\left(n\right)/\mathrm{loglog}\left(n\right),$$

(7) $$\Rightarrow p\geq \sqrt{\frac{\log\left(n\right)}{\left|N\left(v_{i}\right)\right|\mathrm{loglog}\left(n\right)}}.$$

According to Lemma 2, min *p* = $\sqrt{\frac{\log\left(n\right)}{\left|N\left(v_{i}\right)\right|\mathrm{loglog}\left(n\right)}}$ can be taken as a value of the key probability. Therefore, we have *p*_c_’ = $\sqrt{\frac{\log\left(n\right)}{\left|N\left(v_{i}\right)\right|\mathrm{loglog}\left(n\right)}}$. As a result, the relay probability *p*_i_ of a node *v*_i_ can be formulated as:

(8) $$p_{i}=p_{i}’+a=\sqrt{\frac{\log\left(n\right)}{\left|N\left(v_{i}\right)\right|\mathrm{loglog}\left(n\right)}}+(e_{i}-\mathrm{avg})/\mathrm{avg}.$$

**Theorem** **1.** *In an LDC-WSN with n nodes, if n is large enough and each node v_i_ rebroadcasts a packet with probability p_i_ =* $\sqrt{\frac{\log\left(n\right)}{\left|N\left(v_{i}\right)\right|loglog\left(n\right)}}+(e_{i}-\mathrm{avg})/\mathrm{avg}$ *when it receives the packet for the first time, all nodes in the network can receive the packet with a probability near 1, when the process of dissemination completes.*

**Proof**. Combining Lemma 1 and Lemma 2, the conclusion is obvious. □

Based on Theorem 1, we design a function Transmission(*packet*(*v*_j_)) for each node *v*_i_ to relay a received packet *packet*(*v*_j_), as shown in Algorithm 1. Note that the function can also be used for relay short messages. 

**Algorithm 1.** Function Transmission (*packet*(*v*_j_))
1 if (*packet*(*v*_j_) is the first time received by *v*_i_)
2  temp = rand(1);
3  If (temp ≤ *p*_i_) or (*v*_i_ == *v*_j_)
4   *T*(*v*_i_) = *N*(*v*_i_); // *N*(*v*_i_) is the set of *v*_i_’s neighbours
5    While *T*(*v*_i_)! = {}
6      Wake up for one time slot when a node *v*_k_ in *T*(*v*_i_) wakes up;
7      Send the message *packet*(*v*_j_) to *v*_k_;
8      If receives a message ACK(*v*_k_) from *v*_k_
9       Remove *v*_k_ from *T*(*v*_i_);
10      End
11   End
12  End
13 End

In Algorithm 1, when a node *v*_i_ receives a packet *packet*(*v*_j_) for the first time, it will decide whether to rebroadcast it with probability *p*_i_ or not. Moreover, the node *v*_i_ will surely broadcast the packet if it is a source node, i.e., *v*_i_ == *v*_j_. If *v*_i_ decides to relay the packet, it firstly saves the set of its neighbors *N*(*v*_i_) into a set of neighbors that do not receive the packet *T*(*v*_i_). If *T*(*v*_i_) is not empty, *v*_i_ would wake up at the time that the first node *v*_k_ in *T*(*v*_i_) wakes up and sends the packet to *v*_k_. If *v*_i_ receives *v*_k_’s ACK message, it deletes *v*_k_ from *T*(*v*_i_) and then waits for the current first node in *T*(*v*_i_) to wake up and then sends the packet to it. If *v*_i_ does not receive *v*_k_’s ACK message at an expected time period, *v*_i_ will continue to wait for the second node in *T*(*v*_i_) to wake up and then sends the packet to it. The above process will continue until *T*(*v*_i_) is empty. 

### 4.3. Data Storage Algorithm

We will use LT codes to preserve the data. Therefore, we will firstly introduce LT codes, and then present the design of the data storage algorithm.

#### 4.3.1. LT Codes

LT codes [25] are a class of rateless erasure codes, and they are the practical implementation of fountain codes. Given *k* source data {*x*_1_, *x*_2_, …, *x*_k_}, LT codes can produce infinite encoded data {*y*_1_, *y*_2_,….}. LT codes have low encoding and decoding complexities, which benefits them for being applied in sensor networks in which the nodes only have limited computation ability. In LT codes, a probability distribution *Ω*(*j*) is used to produce an encoded degree *d*_m_ for each encoded data *y*_m_, i.e., *y*_m_ is the combination of *d*_m_ source data by Xor operations, where 1 ≤ *j* ≤ *k*, 1 ≤ *d*_m_ ≤ *k*. In the process of decoding, all source data can be recovered with probability 1 − *δ* by using *k* + *ε* encoded data, where *ε* = $O\left(\sqrt{k}ln^{2}\left(\frac{k}{\delta }\right)\right)$, 0 < *δ* < 1.

In LT codes, Robust Soliton distribution is often used as *Ω*(*j*). Its definition is shown as follows. First, an Ideal Soliton distribution is defined:

(9) $$\Omega_{\mathrm{is}}\left(j\right)=\{\begin{array}{c} \frac{1}{k},\;\;\;\;\;\;\;\;\;\;\;\;\;\;\;\;\;\;\;\;\;\;\;\;\;\;\;\;\;\;\;\;\;\;\;j=1\\ \frac{1}{j\left(j-1\right)},\;\;\;\;\;\;\;\;\;\;\;\;\;\;\;j=2,3,\ldots ,k \end{array}.$$

Then, a variable $R=c\ln\left(\frac{k}{\delta }\right)\sqrt{k}$ is used to produce a function τ(j) as follows, where *c* > 0 is a constant:

(10) $$\tau \left(j\right)=\{\begin{array}{c} \frac{R}{jk},\;\;\;\;j=1,\ldots ,\frac{k}{R}-1\\ R\frac{\ln\left(\frac{R}{\delta }\right)}{k},\;\;\;\;\;\;\;\;\;\;\;\;\;\;\;j=\frac{k}{R}\\ 0,\;\;\;\;\;\;\;\;\;j=\frac{k}{R}+1,\ldots ,k \end{array}.$$

Finally, the Robust Soliton distribution can be presented as:

(11) $$\Omega_{\left(j\right)}=\frac{\tau \left(j\right)\;+\Omega_{\mathrm{is}}\left(j\right)}{\beta },\;j=1,2\ldots k,$$

where $\beta =\sum_{j=1}^{k}\left(\tau \left(j\right)+\Omega_{\mathrm{is}}\left(j\right)\right)$.

#### 4.3.2. Algorithm Description

According to the rules of LT codes, each node *v*_i_ firstly computes an encoded degree *d*_i_. When *v*_i_ receives a packet for the first time, it will store the data with probability *d*_i_/*k*. If *v*_i_ decides to store the data, it will combine the data with the encoded data that has been stored in its memory by using an XOR operation, i.e., each node would only store one encoded piece of data. The algorithm of data storage is shown in Algorithm 2. 

**Algorithm 2.** Data Storage Algorithm Ran on Each Node *v*_i_:1 **Upon *v*_i_ generates a source data *x*_i_:**
2  *y*_i_ = *x*_i_;
3  Put *x*_i_ into a message *packet*(*v*_i_); // the message *packet*(*v*_i_) is generated by *v*_i_, and *v*_i_ takes the message as a new message that is received by it for the first time.
4  Transmission(*packet*(*v*_i_));
5
**6 Upon *v*_i_ receives a message *packet*(*v*_j_) that contains a data *x*_j_:**
7  If *packet*(*v*_j_) is received by *v*_i_ for the first time
8   *temp* = rand(1);
9   If (*temp* ≤ *d*_i_/*k*)
10      *y*_i_ = *y*_i_ XOR *x*_j_;
11   End
12   Transmission(*packet*(*v*_j_));
13 End

In Algorithm 2, if a node *v*_i_ is a source node, it will store its data directly. Then, *v*_i_ puts its data into a packet *packet*(*v*_i_). Since *v*_i_ receives the packet for the first time, it will use the function Transmission (*packet*(*v*_i_)) to send the packet to its neighbors. If *v*_i_ is not a source node and receives the packet for the first time, it will firstly store the data with probability *d*_i_/*k*, and then use the function Transmission (*packet*(*v*_i_)) to send the packet to its neighbors.

**Theorem** **2.** *In an LDC-WSN with n nodes and k source data, if n is large enough and every node uses our algorithms to disseminate and store the data, a mobile sink can recover all the source data with probability 1 − δ after it visiting k + ε nodes, where ε =* $O\left(\sqrt{k}ln^{2}\left(\frac{k}{\delta }\right)\right)$*, 0 < δ < 1.*

**Proof.**  According to Theorem 1, each node can receive all the data with probability near 1. When a node receives a data for the first time, it will store the data with probability *d*_i_/*k*. *d*_i_ is got by using the Robust Soliton distribution. Therefore, the encoding degree of the encoded data stored in the node is in coincidence with the requirement of LT codes, i.e., the source data can be recovered with probability 1 − *δ* by using *k* + *ε* encoded data. On the other hand, each node only stores encoded data. Therefore, a mobile sink can recover all the source data with probability 1 − *δ* after it visits *k* + *ε* nodes, according to the feature of LT codes, as shown in Section 4.3.1. □

### 4.4. Performance Analyses

Since data preservation is mainly performed in the second phase of each round, we analyze several key performances of our mechanism in this phase.

#### 4.4.1. Time Complexity

Our mechanism contains two operations: data dissemination and data storage. In data dissemination, each relay node needs to wake up at the time units that its neighbors wake up to transmit data. The number of neighbors for each node is *O*(log*n*) [19]. At the worse situation, a node needs to relay all the source data. Therefore, the worse time complexity of data transmission in a node is *O*(*k*log*n*). In data storage, a node will perform XOR operations on the data received for the first time, which needs at most *O*(*k*) time. Therefore, the time complexity of data storage in a node is *O*(*k*). As a result, the time complexity of our mechanism is *O*(*k*log*n* + *k*).

#### 4.4.2. Energy Consumption

The main energy consumption of data preservation is the process of data dissemination. We proposed a new probabilistic broadcast mechanism, by which only a small proportion of nodes need to relay the data and the other nodes only need to receive the data. Considering the effect of unreliable links, each relay node needs to wake up *O*(|*N*(*v*_i_)|) times to send its data to the neighbors when there is a piece of data that needs to be transmitted, and each non-relay-node needs to wake up *O*(1) times to receive the data. Since there are *k* data in the network, a relay node needs to perform at most *O*(*k*|*N*(*v*_i_)|)transmissions, and each non-relay-node needs to receive the data for *O*(*k*) times. Moreover, the size of each data packet is *b* bits, and a node will consume *E*_s_ (or *E*_r_) energy in transmitting (or receiving) 1-bit data. Therefore, the energy consumption of a relay node is *O*(*bk*|*N*(*v*_i_)|*E*_s_), and the energy consumption of each non-relay-node is *O*(*bkE*_r_).

#### 4.4.3. Latency of Data Dissemination

The latency is defined as the time duration from the time unit that the data is disseminated to the time unit that there are no nodes to relay the data. The latency is highly correlated to the latency of the probabilistic broadcast mechanism. In traditional WSN, the data dissemination latency by using probabilistic broadcast is *O*(1/*r*) [26]. However, in LDC-WSN, the nodes would sleep or wake up periodically. A relay node must wait for the wakeup of its neighbors, and, after that, it can perform the transmission. Moreover, due to the unreliability of communication links, a relay node has to wait and retransmit a piece of data in the following working periods if failures happen in the transmission. Since the length of a working period is *T*, the latency of each transmission is *O*(*T*). As a result, the whole latency of data dissemination is *O*(*T/r*).

#### 4.4.4. Decoding Performance in a Small-Scale Network

According to Theorem 1, the sufficient condition that all nodes in the network can receive all of the data is that the scale of the network should be large enough, i.e., *n* is large enough. On the contrary, we can conclude that the probabilistic broadcast cannot guarantee that all nodes receive the data in small-scale networks. However, we can show that our mechanism is still feasible in small-scale networks as follows.

**Theorem** **3.** *In a small-scale LDC-WSN (i.e., n is not large enough), if each node uses our mechanism to complete the dissemination and storage of k source data, a mobile sink can recover all the source data with probability 1 − δ after it visits Ω(k) nodes*.

**Proof.**  In a small-scale LDC-WSN, some nodes only receive parts of the source data after the data dissemination. Therefore, the encoded data’s actual encoded degrees in these nodes are not equal to the theoretical encoded degrees computed by Robust Soliton distribution. Next, we first analyze the distribution of actual encoded degrees for all of the nodes in the network, and then deduce the decoding performance of our mechanism. □

Assume *A* and *B* are two random variables, and they represent the actual encoded degrees and theoretical encoded degrees of nodes, respectively. *p*’ is the probability that a node receives an arbitrary data packet. Since some nodes may not receive some packets, *p*’ < 1. The probability that a node *v*_i_ achieves an encoded degree of *d*_i_’ is:

(12) $$\mathrm{Pr}(A=d_{i}^{\prime }|B=d_{i})=\left(\begin{array}{c} k\\ d_{i}^{\prime } \end{array}\right){p^{\prime }}^{d_{i}^{\prime }}{\left(1-p^{\prime }\right)}^{k-d_{i}^{\prime }}.$$

Therefore, the actual encoded degree is:

$$\mathrm{Pr}\left(A=d_{i}^{\prime }\right)=\sum_{d_{i}=1}^{k}\mathrm{Pr}\left(B=d_{i}\right)\mathrm{Pr}(A=d_{i}^{\prime }|B=d_{i})$$

(13) $$=\sum_{d_{i}=1}^{k}Ω\left(d_{i}\right)\left(\begin{array}{c} k\\ d_{i}^{\prime } \end{array}\right){p^{\prime }}^{d_{i}^{\prime }}{\left(1-p^{\prime }\right)}^{k-d_{i}^{\prime }}.$$

We use *Ψ*(*j*) = $\mathrm{Pr}\left(A=d_{i}^{\prime }\right)$ to represent the distribution of actual encoded degrees. Then, we set a variable *C_ij_* to represent whether data *x*_j_ is received by a node *v*_i_ or not, i.e., *C_ij_* = 1 means that *x*_j_ is received by *v*_i_, and *C_ij_* = 0 means that it is not received. For each node *v*_i_, we have:

$$\mathrm{Pr}\left(C_{ij}=1\right)=\sum_{d_{i}=1}^{k}\mathrm{Pr}\left(B=d_{i}\right)\mathrm{Pr}(C_{ij}=1|B=d_{i}),$$

(14) $$=\sum_{d_{i}=1}^{k}\Psi \left(d_{i}\right)d_{i}/k=\frac{\sum_{d_{i}=1}^{k}\Psi \left(d_{i}\right)d_{i}}{k}=E\prime \left(d_{i}\right)/k,$$

where $E\prime \left(d_{i}\right)$ is the expected encoded degree according to *Ψ*(*j*) distribution. Since some nodes may not receive some data, we have $E\prime \left(d_{i}\right)$ ≤ $E_{\Omega }\left(d_{i}\right)$, where $E_{\Omega }\left(d_{i}\right)$ is the expected encoded degree according to Robust Soliton distribution. Since $E_{\Omega }\left(d_{i}\right)\leq \sum_{i=2}^{k+1}\frac{1}{i-1}+\sum_{i=1}^{\frac{k}{R}-1}\frac{R}{k}+\ln\left(\frac{R}{\delta }\right)$ [25], $E\prime \left(d_{i}\right)\;\leq \sum_{i=2}^{k+1}\frac{1}{i-1}+\sum_{i=1}^{\frac{k}{R}-1}\frac{R}{k}+\ln\left(\frac{R}{\delta }\right)$. Combined with Formula (14), we have:

(15) $$\;\mathrm{Pr}\left(C_{ij}=1\right)\leq \left(\sum_{i=2}^{k+1}\frac{1}{i-1}+\sum_{i=1}^{\frac{k}{R}-1}\frac{R}{k}+\ln\left(\frac{R}{\delta }\right)\right)/k.$$

We set another variable *D*_j_ to represent whether a source data *x*_j_ can be recovered after the mobile sink vising *m* nodes, i.e., *D*_j_ = 1 means yes, and *D*_j_ = 0 means no. If *x*_j_ cannot be recovered, it means that all of the *m* node does not receive the data before. The probability of this situation is:

$$\mathrm{Pr}\left(D_{j}=0\right)=\prod_{i=1}^{m}\mathrm{Pr}\left(C_{\mathrm{ij}}=0\right),$$

$$=\prod_{i=1}^{m}(1-\mathrm{Pr}\left(C_{\mathrm{ij}}=1\right)),$$

(16) $$\geq {\left(1-\frac{\sum_{i=2}^{k+1}\frac{1}{i-1}+\sum_{i=1}^{\frac{k}{R}-1}\frac{R}{k}+\ln\left(\frac{R}{\delta }\right)}{k}\right)}^{m}.$$

We set *F* to be the event that *k* source data can be recovered after the mobile sink visiting *m* nodes, and we have:

$$\mathrm{Pr}(F)=\prod_{j=1}^{k}\left(1-\mathrm{Pr}\left(D_{j}=0\right)\right),$$

(17) $$\leq {\left(\left(1-{\left(1-\frac{\sum_{i=2}^{k+1}\frac{1}{i-1}+\sum_{i=1}^{\frac{k}{R}-1}\frac{R}{k}+\ln\left(\frac{R}{\delta }\right)}{k}\right)}^{m}\right)\right)}^{k}\;\;\;\;\;.$$

If we need all of the source data to be recovered with probability 1 − *δ*, there are:

$$1-\delta <\mathrm{Pr}\left(F\right)\leq {\left(\left(1-{\left(1-\frac{\sum_{i=2}^{k+1}\frac{1}{i-1}+\sum_{i=1}^{\frac{k}{R}-1}\frac{R}{k}+\ln\left(\frac{R}{\delta }\right)}{k}\right)}^{m}\right)\right)}^{k},$$

(18) $$\Rightarrow \ln(1-\delta )\leq k\ln\left(\left(1-{\left(1-\frac{\sum_{i=2}^{k+1}\frac{1}{i-1}+\sum_{i=1}^{\frac{k}{R}-1}\frac{R}{k}+\ln\left(\frac{R}{\delta }\right)}{k}\right)}^{m}\right)\right).$$

If θ < 1, we have ln(1 − *θ*)=$-\sum_{n=1}^{\infty }\frac{\theta^{n}}{n}$ ≈ −*c*_i_*θ*, where *c*_i_ is a constant. Therefore,

$$-c_{i}\delta \leq -{\mathrm{kc}}_{2}{\left(1-\frac{\sum_{i=2}^{k+1}\frac{1}{i-1}+\sum_{i=1}^{\frac{k}{R}-1}\frac{R}{k}+\ln\left(\frac{R}{\delta }\right)}{k}\right)}^{m},$$

$$\Rightarrow {\left(1-\frac{\sum_{i=2}^{k+1}\frac{1}{i-1}+\sum_{i=1}^{\frac{k}{R}-1}\frac{R}{k}+\ln\left(\frac{R}{\delta }\right)}{k}\right)}^{m}\geq \frac{c_{1}\delta }{c_{2}k},$$

$$\Rightarrow m\ln\left(1-\frac{\sum_{i=2}^{k+1}\frac{1}{i-1}+\sum_{i=1}^{\frac{k}{R}-1}\frac{R}{k}+\ln\left(\frac{R}{\delta }\right)}{k}\right)\geq \ln\frac{c_{1}\delta }{c_{2}k},$$

(19) $$\Rightarrow m\geq \frac{\ln\frac{c_{1}\delta }{c_{2}k}}{\ln\left(1-\frac{\sum_{i=2}^{k+1}\frac{1}{i-1}+\sum_{i=1}^{\frac{k}{R}-1}\frac{R}{k}+\ln\left(\frac{R}{\delta }\right)}{k}\right)}\approx \frac{c_{3}k}{\sum_{i=2}^{k+1}\frac{1}{i-1}+\sum_{i=1}^{\frac{k}{R}-1}\frac{R}{k}+\ln\left(\frac{R}{\delta }\right)}\approx c_{4}k.$$

As a result, *m* = Ω(*k*). 

## 5. Simulations

We developed a simulation platform by using Matlab 7 to test the performances of our mechanism and existing mechanisms. In our simulations, the field of the network is set to be 100 *m* × 100 *m. n* nodes are randomly distributed in the field. The communication radius of the nodes is *r* = 25. In a round, the working time of the network is divided into periods, and each working period *T* contains *m* = 100 time units. Each node only wakes up at one time unit that is randomly chosen by it, and it would sleep in the other time units, except that it needs to send packets to its neighbors. The communication links among the nodes are unreliable [27,28]. The communication successful ratio of each link is selected among the section (0,1) randomly. We mainly simulate the process of data dissemination and storage in the second phase of a data collection round, since this process is the main operation of data preservation. Assuming that there are *k* = 0.1*n* source nodes, they will disseminate their data to the network for preservation. The size of data packet is 1000 bits. The unit energy consumption of sending 1-bit data is *E*_t_ = 100 nJ/bit, and the unit energy consumption of receiving 1-bit data is *E*_r_ = 50 nJ/bit [29]. Since the network works in rounds, we mainly test the performances in a round. 

Typical data preservation mechanisms such as EDFC, DDSLT, P-STCNC and DEC-EaF are selected to compare with ours. We will compare the performances of successful decoding probability and latency.

**Definition** **1**. Successful decoding probability means that the probability that all of the *k* source data can be recovered from the collected encoded data.

**Definition** **2**. Decoding ratio is the ratio that measures the amount of collected encoded data relative to *k*.

**Definition** **3**. Latency is defined as the number of working periods when the process of data preservation completes.

### 5.1. Comparison of Decoding Performance

**Figure 1:**
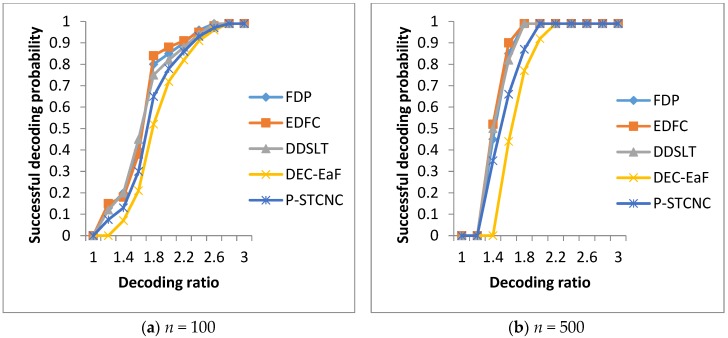
Comparison of decoding performance.

We will test the decoding performances of the mechanisms in two networks with *n* = 100 and *n* = 500, respectively. The simulation results are shown in Figure 1. 

In Figure 1, we can see that FDP, EDFC and DDSLT achieve almost the same successful decoding probability. This is because all three of the mechanisms use LT codes to preserve the data, and they can guarantee that all of the source data are received by all nodes for encoding. Moreover, we can see that the three mechanisms can achieve a successful decoding probability that nears 1 when the decoding ratio is around 2.8 in the network with *n* = 100 nodes and *k* = 10 source data, as shown in Figure 1a. Moreover, the three mechanisms can achieve a successful decoding probability that nears 1 when the decoding ratio is around 1.8 in the network with *n* = 500 nodes and *k* = 50 source data, as shown in Figure 1b. This is because all of the source data can be recovered by using *k* + *ε* encoded data in LT codes. As a result, although the numbers of nodes and source nodes increase largely, all of the source data can be recovered by only using a few more encoded data. 

On the other hand, the three mechanisms achieve higher successful decoding probability than P-STCNC and DEC-EaF. This is because they are optimized to adapt to the sleeping feature of nodes in LDC-WSN and can disseminate all the source nodes to other nodes in the network for effective storage.

### 5.2. Comparison of Energy Consumption and Latency

**Figure 2:**
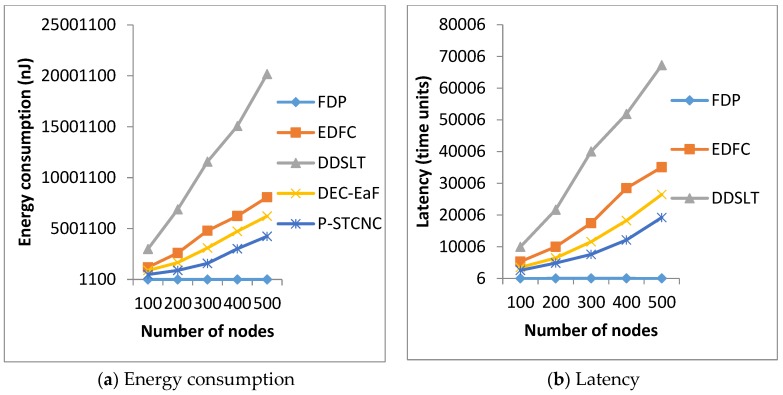
Comparison of energy consumption and latency.

We will test the performances of energy consumption and latency in the networks with *n* = 100, 200, 300, 400 and 500, respectively. The simulation results are shown in Figure 2.

In Figure 2, we can see that FDP achieves much lower energy consumption and latency, compared with the other four mechanisms. In Figure 2a, FDP’s energy consumption is only 0.03%, 0.01%, 0.07% and 0.04% of the energy consumptions of EDFC, DDSLT, P-STCNC and DEC-EaF, respectively. Therefore, FDP has much better energy saving performance. In Figure 2b, FDP’s latency is only 0.04%, 0.02%, 0.05% and 0.08% of the latencies of EDFC, DDSLT, P-STCNC and DEC-EaF, respectively. Therefore, FDP has much better latency performance. The reason why FDP is much better than the four other mechanisms is that it adopts a new probabilistic broadcast to disseminate the data, which is much faster and needs much fewer relay nodes than that of the four other mechanisms. 

## 6. Conclusions

In this paper, we research the problem of how to design a fast and energy-efficient data preservation mechanism for LDC-WSN. In order to prevent the data loss caused by node destruction in harsh environments, the source nodes would disseminate their data to other nodes in the network for preservation as quickly as possible. However, the nodes would sleep for a long time in LDC-WSN, which produces large latency. Moreover, the nodes only have limited memory, so they cannot store all the data. In order to solve these problems, we proposed a probabilistic broadcast and LT codes based data dissemination and storage mechanism. The mechanism is totally distributed, i.e., it does not need the support of global information. Moreover, it can disseminate all the data to the whole network for encoding and storing with low latency. After the data preservation, a mobile sink can enter the network from anywhere and at any time. It can recover all the source data after visiting a few nodes that are still survived. Theoretical analyses and simulations results show that our mechanism has a high successful decoding ratio, and has much lower energy consumption and latency than existing works. 

In the future, we plan to consider a node’s mobility in the networks. The mobility would seriously affect the connectivity of the networks and bring new challenges for effectively disseminating data with low latency. Moreover, data update should be considered in the process of data preservation, which allows new data to be disseminated and stored in time.
